# MicroRNA-940 promotes tumor cell invasion and metastasis by downregulating ZNF24 in gastric cancer

**DOI:** 10.18632/oncotarget.4456

**Published:** 2015-06-27

**Authors:** Xinyang Liu, Xiaoxiao Ge, Zhe Zhang, Xiaowei Zhang, Jinjia Chang, Zheng Wu, Wenbo Tang, Lu Gan, Menghong Sun, Jin Li

**Affiliations:** ^1^ Shanghai Medical College, Fudan University, Shanghai, 200032, P.R. China; ^2^ Department of Medical Oncology, Fudan University Shanghai Cancer Center, Shanghai, 200032, P.R. China; ^3^ Department of Pathology, Fudan University Shanghai Cancer Center, Department of Oncology, Shanghai Medical College, Fudan University, Shanghai, 200032, P.R. China; ^4^ Department of Medical Oncology, Shanghai Tianyou Hospital of Tongji University, Shanghai, 200032, P.R. China

**Keywords:** gastric cancer, miR-940, ZNF24, metastasis, epithelial-mesenchymal transition

## Abstract

Growing evidence indicates that microRNA (miRNA) plays a vital role in progression and metastasis of gastric cancer (GC). However, the underlying mechanism of miRNA-mediated metastasis has not been fully understood. Recently, miRNA-940 (miR-940) was found to be overexpressed in GC, which correlated with malignant progression and poor survival. Mechanistically, we found that miR-940 promoted GC cell migration, invasion, and metastasis *in vivo* by directly and functionally repressing the expression of Zinc Finger Transcription Factor 24 (ZNF24). Importantly, upregulation of ZNF24 could re-inhibit miR-940-induced migration and invasion. Hence, we demonstrated the oncogenic role of miR-940 in GC, finding that miR-940 promoted GC progression by directly downregulating ZNF24 expression, and targeting miR-940 could serve as a novel strategy for future GC therapy.

## INTRODUCTION

Gastric cancer (GC) is the fourth most common cancer and second leading cause of cancer-related deaths worldwide. There are about 700,000 deaths annually [1], more than half of which occur in Asia. Surgical resection remains the primary curative treatment for GC [2]. However, many patients are diagnosed at an advanced stage with extensive invasion and lymphatic metastasis, and most develop regional or distant recurrences after resection. Even with advanced systematic therapy, the 5-year overall survival for patients with late-stage GC was no more than 20% [3]. Therefore, increasing efforts have been devoted to investigating the mechanisms of GC progression, especially the mechanisms of GC metastasis.

MicroRNAs (miRNAs) are small noncoding RNAs that target diverse cellular processes including cell cycle regulation, signaling transduction, transcription regulation, and epigenetic modification [4]. Cumulative evidence suggests that miRNAs contribute to carcinogenesis, tumor progression, and metastasis by serving as oncogenes or tumor suppressive genes [5]. For example, miRNA (miR)-847 [6] and miR-329 [7] are involved in metastasis of GC by inhibiting STAT3/vascular endothelial growth factor (VEGF)-A and TIAM1, respectively.

Previous studies have investigated the role of miR-940 in various diseases. Liang et al. demonstrated that miR-940 reduction contributes to human Tetralogy of Fallot development [8]. Moreover, miR-940 was shown to suppress progression of prostate cancer and pancreatic ductal adenocarcinoma by suppressing Migration and invasion enhancer 1 (MIEN1) [9] and MyD88 [10], respectively. Furthermore, miR-940 also contributed to cisplatin resistance in lung cancer by inhibition of MKP1 and MKK4 [11]. Taken together, by targeting different genes under different pathological conditions, miR-940 might exhibit different biological functions and clinical impacts. Therefore, we should investigate the role of miR-940 in a disease-specific manner.

We found in our previous microarray that miR-940 was overexpressed in GC patients with early recurrence, indicating a potential role of miR-940 in GC recurrence. The hypothesis was further confirmed through data analysis from the online database The Cancer Genome Atlas (TCGA). However, the biological roles and target genes of miR-940 in GC metastasis need further exploration.

In this study, we found that miR-940 was overexpressed in human GC tissues and was associated with poor prognosis. *In vitro* and *in vivo* studies showed that miR-940 contributed to GC metastasis through downregulation of a direct target gene Zinc Finger Transcription Factor 24 (ZNF24), a potential metastasis suppressor. To our knowledge, this is the first study to investigate the role of miR-940 in GC and demonstrate the oncogenic role of miR-940. These results provide a clearer understanding of the underlying mechanism of GC and provide additional information about the function of miR-940.

## RESULTS

### miR-940 is overexpressed in GC and associated with poor survival

**Figure 1:**
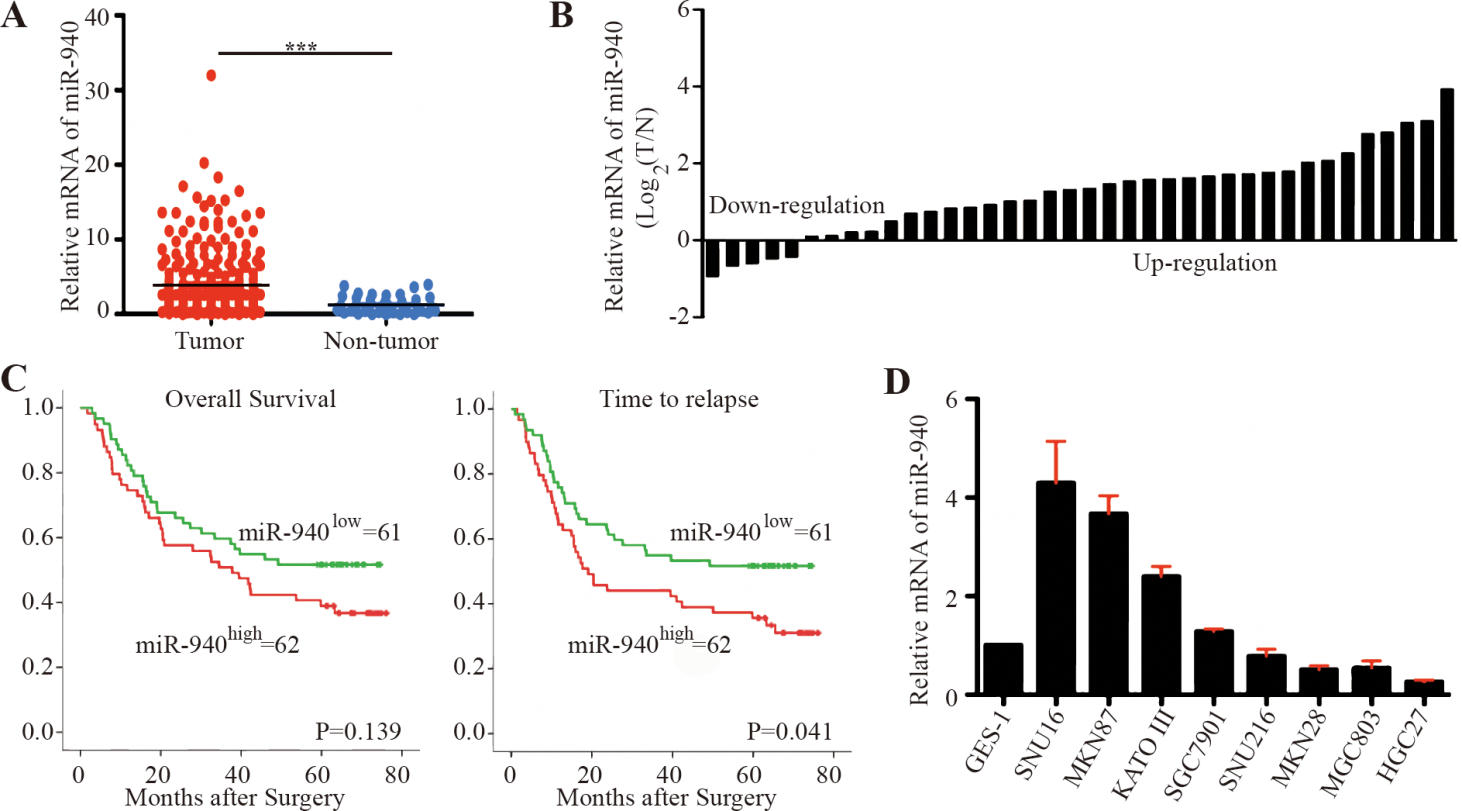
MiR-940 is increased in gastric cancer and associated with poor prognosis **A.** Scatter dot plots show relative mRNA expression of miR-940 in gastric cancer tumor and non-tumor tissues using data from The Cancer Genome Atlas. **B.** Forest plots show miR-940 expression in gastric cancer tumor and corresponding non-tumor tissues. **C.** Kaplan-Meier curves of OS and TTR for gastric caner patients with high/low miR-940 expression. **D.** Relative expression of miR-940 in gastric cancer cell lines. OS, overall survival; TTR, time to recurrence; ***P < 0.001.

To investigate the potential role of miR-940 in GC, the miR-940 expression pattern in GC was evaluated using data from TCGA. The results indicated that a significantly higher level of miR-940 was detected in GC samples than in corresponding non-tumor tissues (Figure 1A). The miR-940 expression in 38 paired GC samples and non-tumor tissues further confirmed this finding (Figure 1B).

In addition, we explored the miR-940 expression in tumor tissues from 123 GC patients (Figure 1C). Consistent with these findings, high expression of miR-940 significantly correlated with advanced N stage (Chi-square *P* = 0.007). The median time to recurrence (TTR) for patients with high miR-940 expression was significantly shorter than for those with low miR-940 expression (log-rank *P* = 0.041). The difference in overall survival (OS) for patients with high and low miR-940 expression failed to reach statistical significance (log-rank *P* = 0.139). Univariate analyses revealed that high expression of miR-940 was significantly associated with decreased TTR (Supplementary Table S1). However, in multivariate Cox proportional hazards regression analysis, adopting all the significant variables in univariate analyses, miR-940 failed to be an independent factor for TTR (Supplementary Table S1).

The expression of miR-940 varied dramatically among GC cell lines (Figure 1D). The normal gastric membrane cell line GES-1 showed relatively low expression of miR-940. Comparatively, MGC803 and HGC27 cells showed lower expression of miR-940, whereas SNU16, MKN87, KATO III, and SGC7901 cells exhibited higher levels of miR-940.

### miR-940 promotes GC cell migration and invasion *in vitro* and metastasis *in vivo*

**Figure 2:**
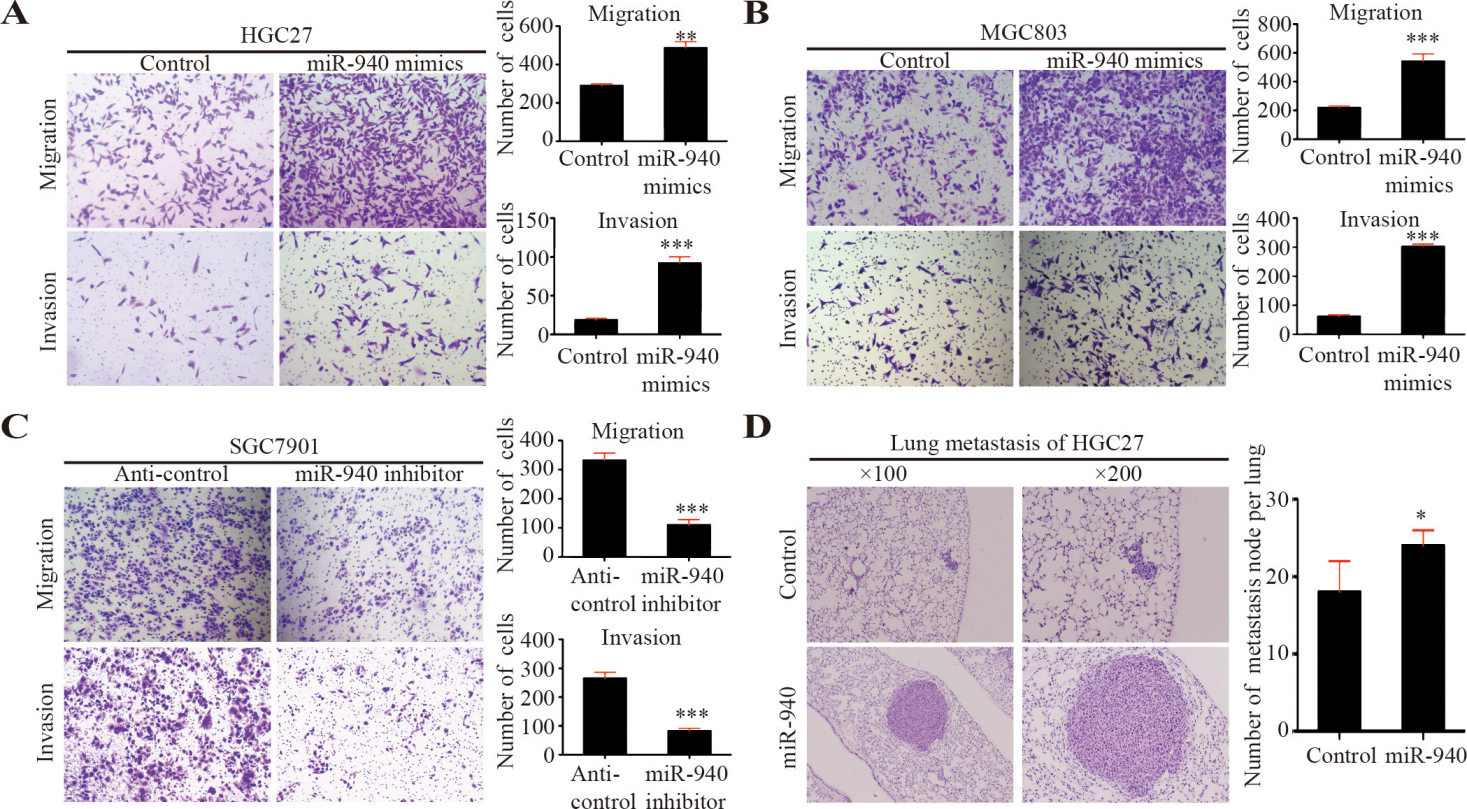
MiR-940 promotes migration and invasion of gastric cancer cells *in vitro* and metastasis *in vivo* **A.** Representative images for HGC27 cells transfected with control/miR-940 mimics in migration and invasion assays. Bar graphs show the statistics for cell counts. **B.** Representative images for MGC803 cells transfected with control/miR-940 mimics in migration and invasion assays. Bar graphs show the statistics for cell counts. **C.** Representative images for SGC7901 cells transfected with anti-control/anti-miR-940 inhibitors in migration and invasion assays. Bar graphs show the statistics for cell counts. **D.** Representative images for HGC27 cells transfected with control/miR-940 mimics in lung metastasis assay *in vivo*. Bar graphs show the statistics for metastatic node counts. *P < 0.05; **P < 0.01; ***P < 0.001.

To further investigate the role of miR-940 overexpression in carcinogenesis and progression of GC, we transfected miR-940 mimics in MGC803 and HGC27 cells, as MGC803 and HGC27 had relative low basal levels of miR-940 in GC cell lines (Figure 1D). Overexpression of miR-940 was confirmed by quantitative real-time polymerase chain reaction (qRT-PCR) (Supplementary Figure S1A). No significant effects were detected in proliferation or colony formation of miR-940 overexpression cells compared with negative control cells (Supplementary Figure S1B and S1F). Overexpression of miR-940 also had no impact on the tumor growth of gastric cancer *in vivo* (Supplementary Figure S1C and S1D). Interestingly, overexpression of miR-940 significantly promoted the migratory and invasive abilities of GC cells (Figure 2A and 2B and Supplementary Figure S1E).

In addition, we transfected miR-940 inhibitors into GC cells, SGC7901 cells, which had relatively high endogenous miR-940 expression (Figure 1D, Supplementary Figure S1A). As expected, we found that knockdown of miR-940 could suppress GC cell migration and invasion (Figure 2C).

To further confirm the role of miR-940 on tumor metastasis *in vivo*, we established miR-940 stably expressing HGC27 cells by lentivirus infection. HGC27 cells stably overexpressing miR-940 were transplanted into NOD-SCID mice through the lateral tail vein along with control cells. Histologic analysis on the lungs of NOD-SCID mice confirmed that miR-940 could also promote lung metastasis of GC *in vivo* (Figure 2D). The number and size of lung metastasis nodules were significantly increased in the HGC27-miR-940 group compared with the control group.

Taken together, the results suggest that miR-940 promotes GC cell migration and invasion *in vitro*, and promotes metastasis *in vivo*.

### miR-940 post-transcriptionally reduces ZNF24 expression by directly binding its three prime untranslated region

**Figure 3:**
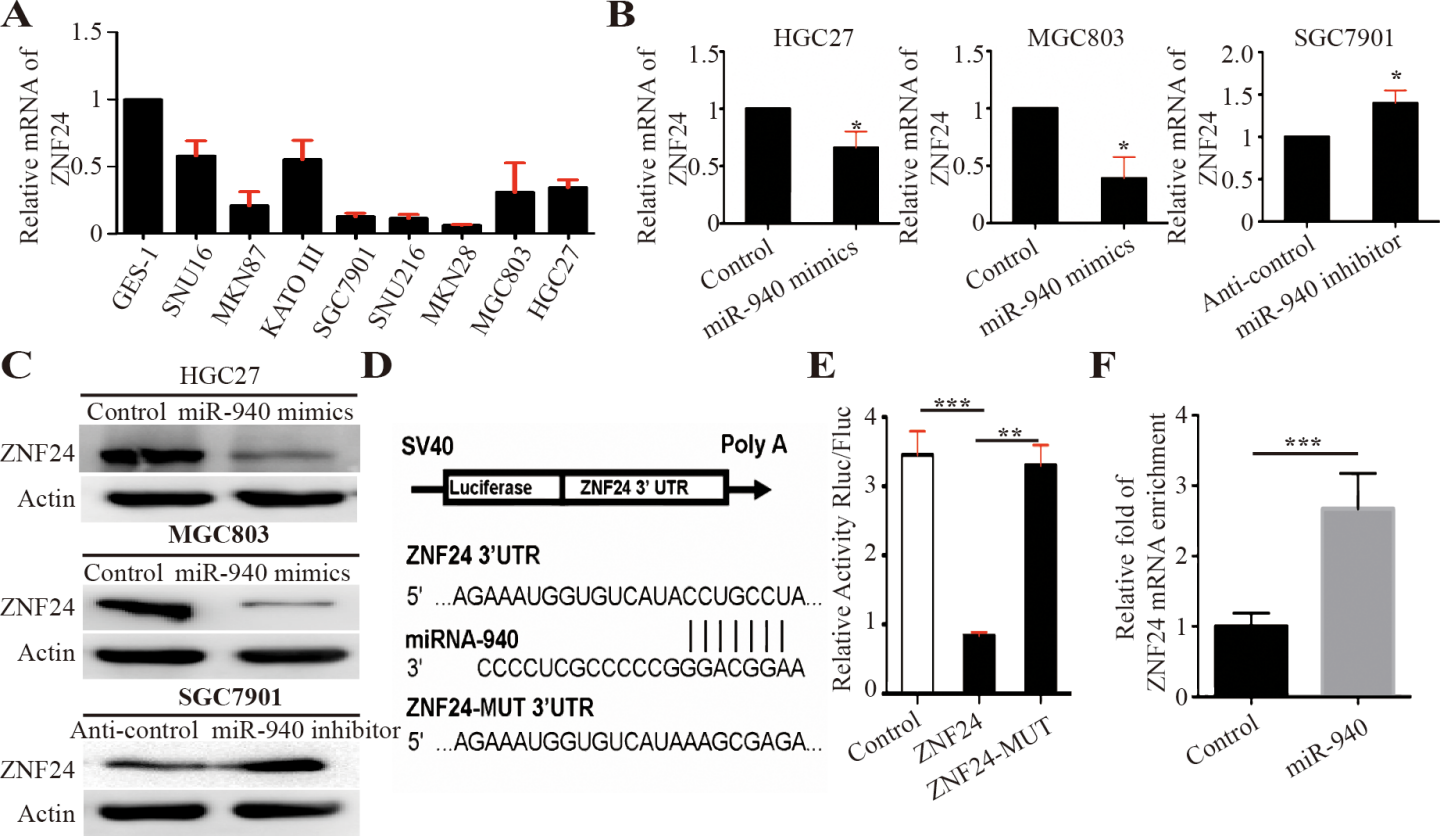
MiR-940 negatively regulates ZNF24 by binding to the ZNF24 3′UTR **A.** Relative mRNA expression of ZNF24 in gastric cancer cell lines. **B.** Relative mRNA expression of ZNF24 in HGC27 cells transfected with control/miR-940 mimics, MGC803 cells transfected with control/miR-940 mimics, and SGC7901 cells transfected with anti-control/anti-miR-940 inhibitors. **C.** Relative protein expression of ZNF24 in HGC27 cells transfected with control/miR-940 mimics, MGC803 cells transfected with control/miR-940 mimics, and SGC7901 cells transfected with anti-control/anti-miR-940 inhibitors. **D.** Illustration shows miR-940-binding site for ZNF24 3′UTR and corresponding mutant binding site. **E.** 3′UTR fragments of ZNF24 and its corresponding mutant counterpart were directly fused to the downstream of the firefly luciferase gene in the luciferase reporter plasmids. Relative luciferase activity was analyzed after the described reporter plasmids (pLuc-3′UTR) or mock reporter plasmid (pLuc) were co-transfected with Renilla plasmid and pWPXL or pWPXL-miR-940 into HEK293T cells. The luciferase activity was normalized to Renilla luciferase activity. The normalized luciferase activity of pLuc group was set as 1. **F.** HEK-293T cells were transfected with miR-940 mimics or control mimics. After 24 h of incubation, cells were harvested and RIP assays were carried out with Argonaute antibody. RNA was obtained and converted to cDNA for qPCR assay to detect the enrichment of ZNF24. *P < 0.05, **P < 0.01; ***P < 0.001.

To further investigate the underlying molecular mechanism of miR-940 in GC metastasis, we used TargetScan [12], miRanda [13], and PicTar [14] to anticipate putative protein-coding gene targets of miR-940. Basing on these analyses, eight candidate genes (ZNF24, SEMA3F, NEDD4, CADM2, SCN4A, STMN2, PDS5A, and CS) that were shared in the results from three databases were selected. We performed qRT-PCR and Western blot to validate the expression of the genes. Relatively high ZNF24 expression was also shown in MGC308 and HGC27 GC cell lines (Figure 3A).

ZNF24 was a vital zinc finger transcription factor containing DNA binding domain and played an important role in tumor progression and angiogenesis. In addition, we detected ZNF24 expression in both miR-940-overexpressing and -inhibiting GC cells. Decreased expression of ZNF24 was shown in miR-940-overexpressing GC cells (Figure 3B and 3C). Conversely, increased expression of ZNF24 was detected in knockdown of miR-940 expression in GC cells (Figures 3B and 3C).

To identify whether ZNF24 was a target gene of miR-940, we analyzed the three prime untranslated region (3′UTR) sequence of ZNF24 using TargetScan. The results revealed one potential binding site for miR-940, indicating that ZNF24 gene transcript might be a potential target gene for miR-940 (Figure 3D). To test whether ZNF24 was a direct binding for miR-940, a series of 3′UTR fragments of ZNF24, including full length with binding site, and corresponding mutant counterpart were directly fused downstream of the firefly luciferase gene (pLuc; Figure 3D). The luciferase-3′UTR construct (pLuc-3′UTR) as described was co-transfected into HEK-293T cells together with Renilla plasmid and pWPXL-miR-940 or pWPXL control. The efficiency of transfection was normalized by cotransfection with Renilla reporter vector. As shown in Figure 3E, miR-940 could significantly increase the luciferase activity of the full length ZNF24 3′UTR construct, whereas in the counterpart with the mutant binding site, the luciferase activity was not significantly changed. These data indicate that miR-940 downregulated ZNF24 expression by directly binding its 3′UTR. Furthermore, Ago2 immunoprecipitation (Ago2-IP) assay showed that ZNF24 mRNA was enriched into RISC complex after transfection with miR-940 mimics (Figure 3F), further validating that ZNF24 is a direct target gene of miR-940.

### ZNF24 is involved in miR-940-induced activation of GC cell migration and invasion

**Figure 4:**
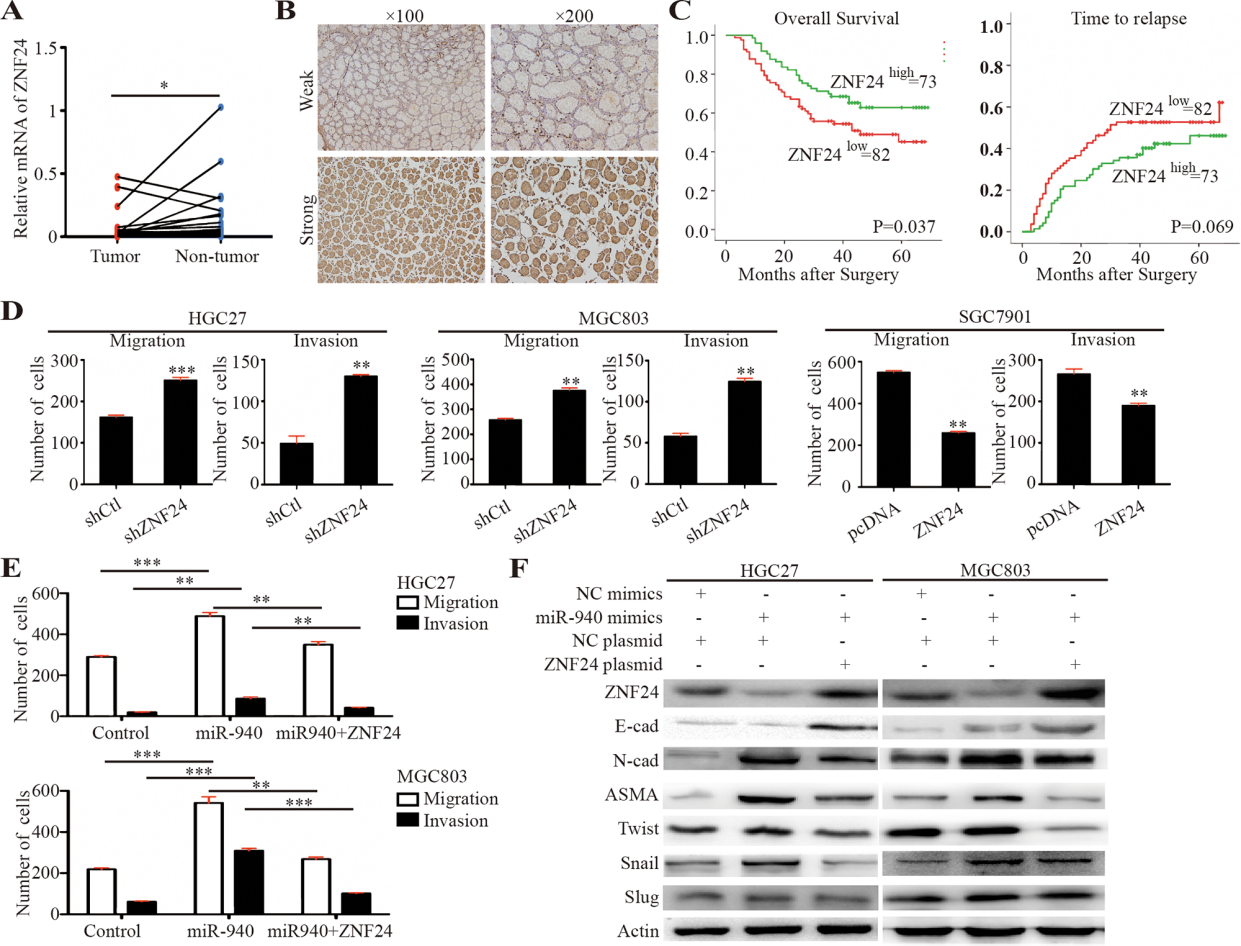
ZNF24 serves as a potential tumor suppressor and is inversely correlated with miR-940 expression in gastric cancer **A.** Scatter dot plots show relative mRNA expression of ZNF24 in gastric cancer tumor and corresponding non-tumor tissues. **B.** Representative images for gastric cancer with weak/strong ZNF24 expression. **C.** Kaplan-Meier curves of OS and TTR for gastric caner patients with weak/strong ZNF24 expression. **D.** Bar graphs show the migration and invasion assays for HGC27 and MGC803 cells transfected with control/shZNF24 plasmids and SGC7901 cells transfected with control/ZNF24 plasmids. **E.** Bar graphs show the migration and invasion assays for HGC27 and MGC803 cells transfected with control/miR-940/miR-940+ZNF24. **F.** Key molecules of EMT were assessed in the indicated cells. OS, overall survival; TTR, time to recurrence; EMT, epithelial-mesenchymal transition. **P < 0.01; ***P < 0.001.

For the first time, ZNF24 was reported to be associated with tumor invasion and metastasis, but the effects of ZNF24 on GC cells have not been established. To explore the functions of ZNF24, miRNA expression of ZNF24 was detected in GC and corresponding non-tumor tissues. A significantly higher level of ZNF24 was detected in non-tumor tissues than in corresponding tumor samples (Figure 4A).

The clinical relevance of ZNF24 was further explored using tissue microarray of 155 patients' samples. ZNF24 showed relatively homogenous staining across each tumor section. Thus, we only measured the staining intensity of ZNF24 in each tumor. ZNF24 immunostaining intensities were semiquantitatively scored as: 0, none; 1, weak; 2, moderate; and 3, intense, by two observers independently. Patients were dichotomized into high (score of 2 or 3) or low (score of 0 or 1) expression of ZNF24 (Figure 4B). Decreased ZNF24 expression significantly correlated with aggressive tumor phenotypes, such as larger tumor size, lymph mode metastasis, and advanced stage (Table 1). The median OS for patients with low ZNF24 expression was 35.5 months, which was significantly shorter than for those with high expression of ZNF24 (median OS, 43.0 months; log-rank *P* = 0.037). Similarly, there was borderline significance between median TTR for patients with low ZNF24 expression (median TTR, 29.0 months) and those with high ZNF24 expression (median TTR, 41.0 months; log-rank *P* = 0.069). Univariate analyses revealed that low expression of ZNF24 was significantly associated with decreased OS and had a borderline significant association with decreased TTR (Figure 4C and Table 2). However, in multivariate Cox proportional hazards regression analysis, adopting all the significant variables in univariate analyses, ZNF24 failed to be an independent factor for OS and TTR (Table 2).

**Table 1:** Clinicopathological characteristics of gastric cancer patients in different ZNF24 expression subgroups

	ZNF24 expression				
	Low		High		
Characteristic	No. of patients	%	No. of patients	%	*P* value
**Total**	82	52.9	73	47.1	
**Age (years)**					0.161
<60	48	58.5	36	49.3	
≥60	34	41.5	37	50.7	
**Sex**					0.102
Female	24	29.3	14	19.2	
Male	58	70.7	59	80.8	
**Site**					**0.096**
Cardia	14	17.1	21	28.8	
Fundus	15	18.3	12	16.4	
Body	50	61.0	38	52.1	
Antrum	0	0.0	2	2.7	
Pylus	3	3.7	0	0.0	
**Differential status**					0.558
Undifferentiated/poorly	70	85.4	62	84.9	
Moderate/well	12	14.6	11	15.1	
**Nerve invasion**					0.117
No	38	46.3	26	35.6	
Yes	44	53.7	47	64.4	
**Vascular invasion**					0.377
No	35	42.7	29	39.7	
Yes	47	57.3	44	60.3	
**T**					**0.021**
T1/2/3	9	11.0	18	24.7	
T4	73	89.0	55	75.3	
**N**					**0.077**
N0/1/2	46	56.1	50	68.5	
N3	36	43.9	23	31.5	
**M**					0.271
M0	73	89.0	68	93.2	
M1	9	11.0	5	6.8	
**Stage**					**0.063**
I/II	20	24.4	27	37.0	
III/IV	62	75.6	46	63.0	

**Table 2:** Univariate and multivariate analysis of factors associated with overall survival and time to recurrence in gastric cancer patients

	Overall survival						Time to recurrence					
	Univariate analysis			Multivariate analysis			Univariate analysis			Multivariate analysis		
	Regression coefficient	HR (95%CI)	*P* value	Regression coefficient	HR (95%CI)	*P* value	Regression coefficient	HR (95%CI)	*P* value	Regression coefficient	HR (95%CI)	*P* value
Age (>60 vs. ≤60 years)	−0.077	0.926 (0.572, 1.499)	0.754				−0.058	0.944 (0.598, 1.489)	0.803			
Sex (male vs. female)	−0.2801	0.755 (0.444, 1.285)	0.301				−0.273	0.761 (0.460, 1.260)	0.289			
Differential status (high/moderate vs. low/undifferentiated)	−0.519	0.595 (0.272, 1.302)	0.194				−0.641	0.527 (0.242, 1.148)	0.107			
Nerve invasion (yes vs. no)	0.384	1.468 (0.886, 2.432)	0.136				0.494	1.639 (1.009, 2.665)	**0.046**	0.234	1.264 (0.765, 2.087)	0.360
Vascular invasion (yes vs. no)	0.088	1.092 (0.668, 1.786)	0.725				0.117	1.124 (0.706, 1.790)	0.621			
T (4 vs. 1/2/3)	0.483	1.62 (0.802, 3.275)	0.179				0.765	2.149 (1.032, 4.477)	**0.041**	0.381	1.976 (0.937, 4.167)	0.074
N (3 vs. 0/1/2)	1.141	3.131 (1.916, 5.116)	**0.000**	1.157	3.182 (1.924, 5.261)	**0.000**	1.164	3.204 (2.016, 5.091)	**0.000**	1.162	3.198 (1.986, 5.149)	**0.000**
M (1 vs. 0)	1.373	3.948 (2.056, 7.579)	**0.000**	1.505	4.505 (2.315, 8.767)	**0.000**	1.440	4.220 (2.248, 7.923)	**0.000**	1.585	4.881 (2.523, 9.443)	**0.000**
ZNF24 (high vs. low)	−0.514	0.598 (0.366, 0.978)	**0.041**	−0.324	0.723 (0.439, 1.192)	0.204	−0.420	0.657 (0.415, 1.041)	**0.073**	−0.262	0.769 (0.481, 1.230)	0.273

HR: hazard ratio; CI: confidence interval.

The biological impacts of ZNF24 on GC cell migration and invasion were also evaluated using *in vitro* assays (Figure 4D and Supplementary Figure 2A). Decreased migration and invasion were both observed in ZNF24 upregulation GC cells, whereas increased impacts were detected in ZNF24 downregulation cells. Taken together, ZNF24 might be a potential tumor suppressor gene in GC.

### miR-940 promotes GC cell invasion and metastasis via epithelial-mesenchymal transition

To further explore the underlying mechanism of enhanced migration and invasion by miR-940 overexpression, epithelial-mesenchymal transition (EMT) molecules were evaluated. The typical EMT phenotype, including upregulation of N-cadherin and alpha smooth muscle actin (ASMA), was observed in miR-940 overexpression GC cells (Figure 4F). However, no significant E-cadherin expression alterations were observed in both miR-940 overexpression cells. This might partly be due to the low basal E-cadherin expression in these cells. Alterations of EMT molecules were accompanied by a parallel regulation of Snail rather than Slug or Twist, indicating that Snail may correlate with the miR-940-mediated EMT process.

Consistently, ZNF24 played an important role in the miR-940-mediated EMT process. Overexpression of ZNF24 attenuated the EMT prompting effect of miR-940, indicating that miR-940 promoted EMT through interfering ZNF24 (Figure 4F). *In vitro* migration and invasion assays further validated this interaction (Figure 4E and Supplementary Figure 2B). Migration and invasion were significantly compromised when ZNF24 was overexpressed in miR-940-overexpressing cells. Lower levels of N-cadherin, ASMA, and Snail, as well as a higher level of E-cadherin, were detected in ZNF24-miR-940 dual-overexpressing cells compared with miR-940 cells. As shown in Supplementary Figure S3, when ZNF24 was overexpressed, EMT process was suppressed in both cell lines. This result suggested that miRNA-940 might indirectly influence EMT through regulating ZNF24.

## DISCUSSION

Metastasis is a crucial factor in determining the prognosis of GC patients. Therefore, identifying metastatic factors and elucidating the underlying molecular mechanisms become critical issues. Recent studies reveal that several miRNAs play important roles in GC initiation and progression, such as miR-34 [15], miR-500 [16], miR-126 [17], and miR-203 [18]. Identifying these biomarkers could provide new insights for understanding the mechanisms of GC progression, and designing better therapeutic strategies.

Currently, the dysregulation of miR-940 has been found in various cancers [9]. Rajendiran and colleagues found that miR-940 was highly expressed in normal tissues compared with tumors, and miR-940 inhibited migratory and invasive potential of prostate cancer cells and increased E-cadherin expression by regulating MIEN1. Ma et al. [19] reported that miR-940 downregulates Nestin and thus suppresses proliferation of nasopharyngeal carcinoma cells through involvement in the DNA damage response. Wang et al. [11] reported that RIP1 suppresses cisplatin-induced and JNK-mediated cytotoxicity through release of the constraint of miR-940 on MKP1 expression in lung cancer. However, in the study of Hu et al. [20], miR-940 was a candidate for selection of stable endogenous control for quantification of circulating miRNAs in cancer patients (screening and validation: healthy, chronic hepatitis B, cirrhosis, hepatocellular carcinoma, colorectal cancer, lung cancer; application phase: esophageal, gastric, breast, renal, and prostate cancers), which implied that miR-940 expression was relatively stable. The inconsistency among published studies as to the roles and targets of miR-940 in various cancers suggest that it might play different roles in different cancers. As one miRNA usually have several target genes, it may mainly target different genes in different tissues or tumors, according to the genetic and epigenetic expression signatures in different tissues. Thus, the biological functions observed in different tumors are sometimes inconsistent. In addition, some genes can function as both oncogene and tumor suppressing genes depending on the microenvironment. These may be the reasons why miR-940 play different roles in various cancers.

In GC, the function of miR-940 is still unknown. In the present study, we showed that miR-940 was frequently overexpressed in human GC and overexpression of miR-940 was significantly associated with decreased OS and increased recurrence rate. Further studies showed that overexpression of miR-940 promoted GC cell migration and invasion *in vitro* as well as metastasis *in vivo* through the EMT process. As biological function of miRNA was achieved by interfering target genes, we identified that miR-940 could promote GC invasion and metastasis through downregulation of ZNF24, a direct and functional target gene of miR-940. Overexpression of ZNF24 could attenuate the oncogenic role of miR-940 as well the EMT process. Taken together, the data indicated that miR-940 acted as a novel oncogene in GC and served as a potential therapeutic target.

ZNF24, also known as ZNF191 [21] and KOX17 [22], contains four zinc finger motifs that encode putative DNA binding domains. Due to the location of the ZNF24 gene, on chromosome 18q12.1 [22], a region frequently deleted in colorectal carcinoma, it possibly plays a role in the negative regulation of tumor growth [23]. Recently, Jia et al. [24] reported that ZNF24 functioned as a negative regulator of developmental and tumor angiogenesis by direct binding to an 11-bp fragment of the VEGF proximal promoter and therefore inhibited VEGF transcription. These results are consistent with our findings that ZNF24 serves as a tumor suppressor in GC.

In summary, our results show that miR-940 is frequently overexpressed in GCs associated with a poor prognosis. Overexpression of miR-940 promotes GC cell migration and invasion by binding and downregulating the tumor-suppressing gene ZNF24. The results suggest that miR-940 contributes to metastasis and progression of GC and that miR-940 may have therapeutic potential in GC metastasis.

## MATERIALS AND METHODS

### Human samples

123 human GC and 75 adjacent non-tumor tissues were collected from patients who underwent surgical resection at the Department of Gastric Cancer and Soft Tissue Sarcomas, Shanghai Cancer Center of Fudan University, Shanghai, China, from Jan 2008 to Dec 2009 for quantitative PCR analysis. All tissues were immediately frozen in liquid nitrogen and stored at −80°C in a refrigerator.

Paraffin-embedded tumor tissues collected from 155 consecutive patients with GC between Jan 2008 and Dec 2009 were used for tissue microarray construction and immunohistochemistry. Clinical data collection and postoperative follow-up procedures were done according to a uniform guideline of the Shanghai Cancer Center of Fudan University.

Informed consents were obtained from all patients and the study was approved by the Clinical Research Ethics Committee of Fudan University Shanghai Cancer Center.

### Cell culture

Nine human GC cell lines (GES-1, SNU16, N87, KATOIII, SGC7901, SNU216, N28, MGC803, and HGC27) were purchased from the Cell Resource Center, Shanghai Institute of Biochemistry and Cell Bank at the Chinese Academy of Sciences. Cell lines were routinely authenticated by DNA-fingerprinting and isoenzyme analyses and checked for contamination by mycoplasma using Hoechst staining. All cell lines were maintained in Roswell Park Memorial Institute 1640, Minimum Essential Medium, or Dulbecco's Modified Eagle's medium supplemented with 10% fetal bovine serum under a humidified air atmosphere containing 5% carbon dioxide.

### RNA extraction and quantitative real-time PCR

Total RNA from 123 tumors and 75 adjacent non-tumor tissues, and cultured cells was extracted using TRIzol reagent (Invitrogen, Carlsbad, CA, USA). qRT-PCR assays were carried out to detect mRNA expression according to the manufacturer's instructions (Takara, Shiga, Japan). The paired primerzs for ZNF24 were forward primer: “GTTGCCATCCTACCCAAAGA” and reverse primer: “GTTCACTCTCCAAATCCTCCA”. Beta-actin was used as an internal control with forward primer: “CTCCATCCTGGCCTCGCTGT” and reverse primer: “GCTGTCACCTCCACCGTTCC”. The expression levels of mature miRNA-940 were measured by Universal ProbeLibrary (Roche, Basel, Switzerland) according to the manufacturer's instructions, and small nuclear RNA U6 was used as an internal control.

### Lentivirus production and transduction

The miR-940-overexpressing plasmid was purchased from Hanbio (Shanghai, China) and was transfected into HEK293T cells using FuGene^®^ HD Transfection Reagent (Roche) to generate lentivirus. HGC27 cells were infected with the recombinant lentivirus plus 5 μg/mL Polybrene^®^ (Sigma-Aldrich, St Louis, MO, USA).

### Plasmid transfection

The pWPXL-ZNF24 plasmid and shZNF24 plasmid were purchased from Hanbio. The sequence of ZNF24 shRNA was: top “GATCCGGAGGATTTGGAGAGTGAACTTTTCAAG AGAAAGTTCACTCTCCAAATCCTCTTTTTTC”, and bottom “AATTGAAAAAAGAGGATTTGGAGAGT GAACTTTCTCTTGAAAAGTTCACTCTCCAAATCCT CCG”. Plasmid transfection was done using FuGene HD Transfection Reagent according to the manufacturer's protocol.

### Oligonucleotide transfection

miR-940 mimics and inhibitors were synthesized by GenePharma (Shanghai, China). The sequence of miR-940 was: “AAGGCAGGGCCCCCGCUCCCC”. The sequence of the inhibitor was: “GGGGAGCGGGGGCCCUGCCUU”. Oligonucleotide transfection was done using FuGene HD Transfection Reagent according to the manufacturer's protocol.

### Cell proliferation assays

MGC803 and HGC27 cells were seeded at a density of 1000 cells per well in 96-well plates. The cells were transfected with miR-Normal Control (NC) mimics, miR-940 mimics, miR-940 inhibitors, or miR-NC inhibitors. Cell proliferation was analyzed using Cell Counting Kit 8 (Dojindo, Kumamoto, Japan) according to the manufacture's protocol for 4 days.

### Colony formation assays

MGC803 and HGC27 cells were seeded into 6-well plates at a concentration of 5000 cells per well. Cells were cultured for 12–16 days according to the character of each cell line. Then, the cells were fixed with 100% methanol and stained with 0.1% crystal violet.

### Scratch assays

Cells were seeded in a 6-well plate, and a “wounding” line was scratched into the cell monolayer with a sterile 200-μL pipette tip. The width of the wound was measured under a microscope at 0, 24, and 48 hours after the scratch to assess the migration ability of the cells.

### Cell migration and invasion assays

*In vitro* migration and invasion assay were performed in chamber of 8-μm Transwell inserts (BD Falcon™; Becton Dickinson, Franklin Lakes, NJ, USA) with or without Matrigel (BD Falcon™). 20000 MGC803 cells and HGC27 cells and 40000 SGC7901 cells were placed into the top chamber of each insert in serum-free medium and serum-containing medium was used in the lower chamber as the attractant. According to the character of the used cell lines, cells that migrated were fixed and stained in dye solution containing 0.1% crystal violet and 20% methanol after 24–36 hours of incubation at 37°C. The number of cells that had migrated was counted using an IX71 inverted microscope (Olympus Corp, Tokyo, Japan).

### Luciferase assays

HEK293T cells were seeded in 96-well plates at 8000 cells per well the day before transfection. A mixture of 100 ng pLuc-3′UTR, 200 ng pWPXL-940, and 20 ng Renilla plasmid (containing no 3′UTR) was transfected into HEK293T cells with Lipofectamine^®^ 2000 (Life Technologies, Carlsbad, CA, USA) in each well. After 48 hours, firefly and Renilla luciferase activities were measured with a Dual-Luciferase^®^ Reporter Assay System (Promega, Madison, WI, USA). The Renilla luciferase activities were used as an internal control for transfection efficiency.

### RNA-binding protein immunoprecipitation (RIP) assays

HEK-293T cells were cultured in 10 cm-dish plates and transfected with 30 nmol miR-940 mimics or control mimics. After 24 h of incubation, cells were harvested and RIP assays were carried out with Argonaute antibody (Abcam, Cambridge, UK), according to the manufacturer's guidelines (Millipore, Billerica, U.S.A). RNA was obtained and converted to cDNA for qPCR assay to detect the enrichment of ZNF24.

### *In vivo* proliferation and metastasis assays

For proliferation assays, HGC27 cells infected with miR-940-overexpressing lentivirus and corresponding mock lentivirus were transplanted into node mice (4-week-old, 7 per group, 1 × 10^7^ cells for each mouse) subcutaneously. Xenografts were measured twice a week and mice were sacrificed when the largest exnograft exceeded 1000 mm^3^. For metastasis assays, HGC27 cells infected with miR-940-overexpressing lentivirus and corresponding mock lentivirus were transplanted into NOD-SCID mice (4-week-old, 8 per group, 1 × 10^7^ cells for each mouse) through the lateral tail vein. After 8 weeks, mice were sacrificed and their lungs were removed and subjected to hematoxylin and eosin staining. All research involving animals complied with protocols approved by the Shanghai Medical Experimental Animal Care Commission.

### Western blot

Standard Western blot procedures were performed as described previously [25]. The primary antibodies used were shown in Supplementary Table S2. The proteins were visualized with Pierce™ enhanced chemiluminescence reagents (Life Technologies).

### Immunohistochemical staining

Briefly, 4-μm sections were deparaffinized and subjected to antigen retrieval (citrate buffer, pH = 6.0). Sections were then incubated overnight at 4°C with mouse monoclonal antibody to ZNF24 (1:100 dilution; Abnova, Taipei, Taiwan). Reaction products were visualized with 3, 3′-diaminobenzidine tetrahydrochloride and counterstained with hematoxylin and eosin. ZNF24 showed relatively homogenous staining across each tumor section. Thus, we only measured the staining intensity of ZNF24 in each tumor. ZNF24 immunostaining intensities were semiquantitatively scored as: 0, none; 1, weak; 2, moderate; and 3, intense, by two observers independently. In the analysis of IHC results, a score of 2 or 3 is considered “strong” and 0 or 1 is considered “weak”.

### Statistical analysis

Statistical analyses were conducted using the Statistical Package for the Social Sciences version 18.0 (SPSS Inc, Chicago, IL, USA). Analyses of MiRNA-940 expression with clinicopathologic parameters were performed using Fisher's exact test. Variables associated with OS and TTR were identified using univariate Cox proportional hazards regression models. Significant factors in univariate analysis were further subjected to multivariate Cox regression analysis in a stepwise manner. Kaplan-Meier plots (log-rank tests) were used to describe OS and TTR. The results of functional experiments were presented as the means ± standard deviation and evaluated using a Mann-Whitney *U* test. A two-tailed *P* < 0.05 was considered significant.

## SUPPLEMENTAY FIGURES AND TABLES


