# Supplementary material for: MicroRNA-940 promotes tumor cell invasion and metastasis by downregulating ZNF24 in gastric cancer
Source: Oncotarget. 2015 Jun 27;6(28):25418–28. doi: 10.18632/oncotarget.4456 (PMC4694841; doi:10.18632/oncotarget.4456)
Supplement: Supplementary file 1 [file oncotarget-06-25418-s001.pdf]

SUPPLEMENTAY FIGURES AND TABLES

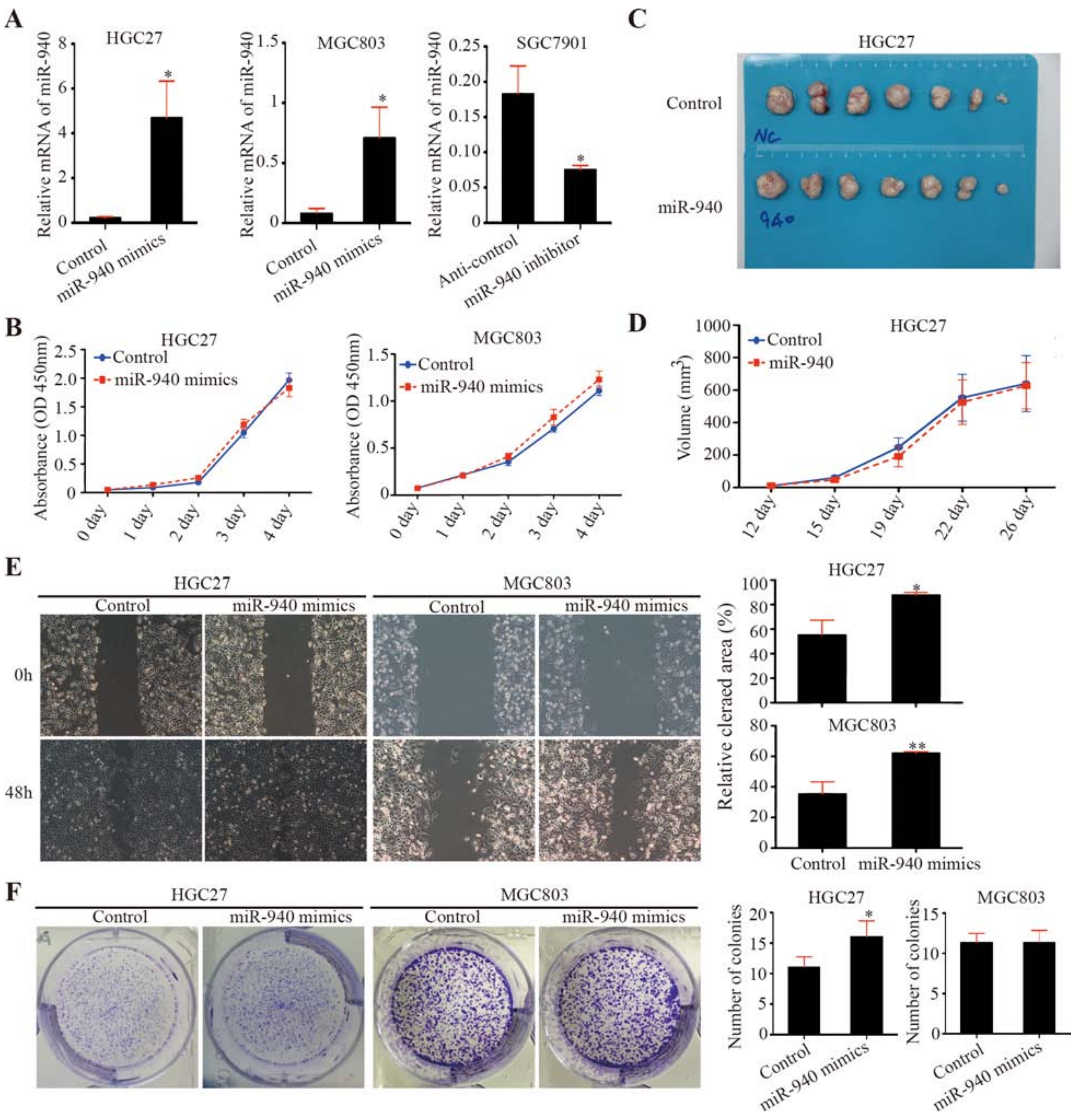

Supplementary Figure S1: Impacts of miR-940 on gastric cell proliferation, migration and colony formation. A. Efficiency validation of transfection of miR-940 mimics and inhibitors. **B.** Proliferation assays for HGC27 and MGC803 cells transfected with control/miR-940 mimics. **C.** Images of xenografts for HGC27 cells transfected with control/miR-940 lentivirus. **D.** *In vivo* proliferation assays of xenografts for HGC27 cells transfected with control/miR-940 lentivirus. **E.** Scratch assays for HGC27 and MGC803 cells transfected with control/miR-940 mimics. Bar graphs show relative cleared area. **F.** Colony formation assays for HGC27 and MGC803 cells transfected with control/miR-940 mimics. Bar graphs show colony count. *$P < 0.05$; **$P < 0.01$.

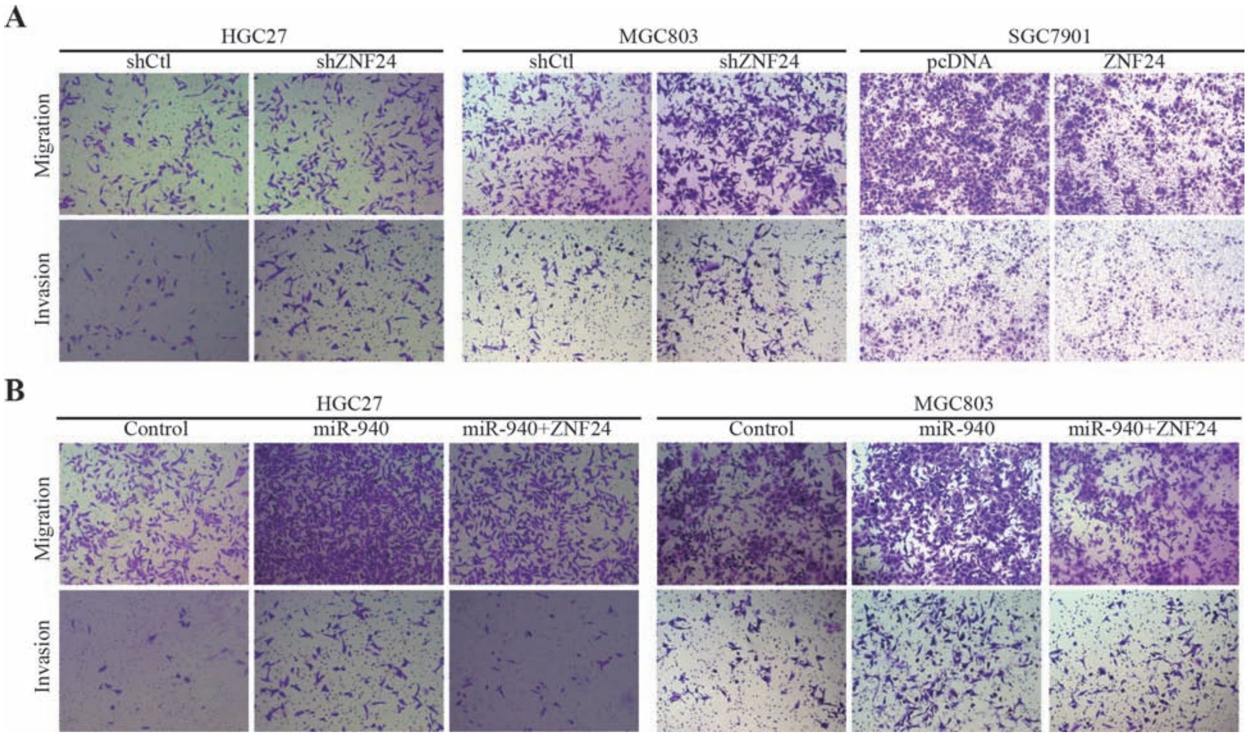

Supplementary Figure S2: ZNF24 supresses migration and invasion of gastric cancer cells *in vitro*. A. Representative images show the migration and invasion assays for HGC27 and MGC803 cells transfected with control/shZNF24 plasmids as well as SGC7901 cells transfected with control/ZNF24 plasmids. **B.** Representative images show the migration and invasion assays for HGC27 and MGC803 cells transfected with control/miR-940/miR-940+ZNF24.

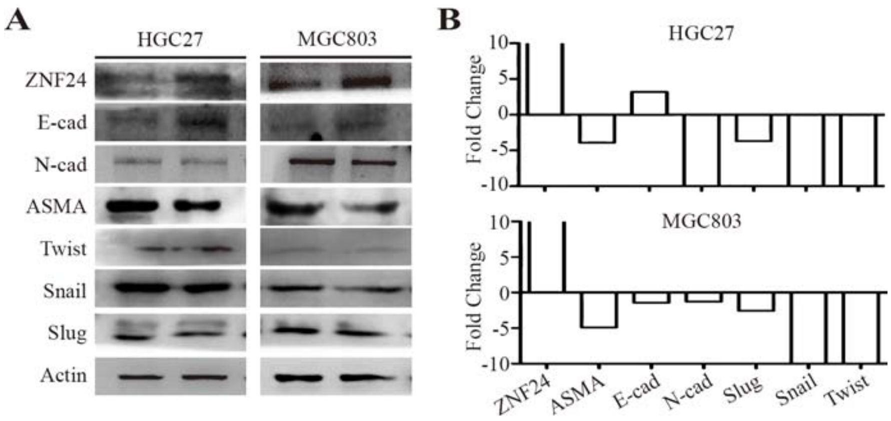

Supplementary Figure S3: miRNA-940 indirectly influence EMT through regulating ZNF24. **A.** Key molecules of EMT were assessed by western blots in the indicated cells. **B.** Key molecules of EMT were assessed by qRT-PCR in the indicated cells.

Supplementary Table S1. Univariate and multivariate analysis of factors associated with time to recurrence in 123 gastric cancer patients

	Time to recurrence					
	Univariate analysis			Multivariate analysis		
	Regression coefficient	HR (95%CI)	*P* value	Regression coefficient	HR (95%CI)	*P* value
Lauren type (diffusal v.s. intestinal)	−0.590	0.554 (0.356, 0.861)	**0.009**	−0.278	0.757 (0.586, 0.977)	**0.033**
Nerve invasion (yes vs. no)	0.834	2.304 (1.432, 3.704)	**0.001**	0.314	1.368 (1.024, 1.828)	**0.034**
Vascular invasion (yes vs. no)	0.437	1.548 (0.960, 2.500)	**0.073**	0.148	1.160 (0.867, 1.550)	0.317
T (4 vs. 1/2/3)	0.702	2.018 (1.111, 3.664)	**0.021**	0.566	1.761 (0.892, 3.479)	**0.034**
N (3 vs. 0/1/2)	0.664	3.204 (2.016, 5.091)	**0.000**	0.058	1.060 (0.625, 2.797)	0.829
M (1 vs. 0)	0.981	1.942 (1.237, 3.049)	**0.004**	0.734	2.083 (1.153, 3.762)	**0.015**
miR-940 (high vs. low)	0.489	1.631 (1.015, 2.619)	**0.043**	0.477	1.611 (0.964, 2.694)	**0.069**

HR: hazard ratio; CI: confidence interval.

Supplementary Table S2. Primary antibodies for Western blot and immunohistochemistry

Antibody	Concentration for WB	Concentrationfor IHC	Specificity	Company
ZNF24	1:1000	1:100	Mouse monoclonal	Abnova
E-cadherin	1:1000	/	Mouse monoclonal	Abcam
N-cadherin	1:1000	/	Rabbit polyclonal	Abcam
Vimentin	1:1000	/	Rabbit monoclonal	Abcam
α-SMA	1:500	/	Rabbit polyclonal	Abcam
Snail	1:200	/	Rabbit monoclonal	Cell Signaling
Slug	1:500	/	Rabbit polyclonal	Abcam
β-actin	1:2000	/	Mouse monoclonal	Sigma

WB: Western blot; IHC: immunohistochemical staining.
